# From appraisal to word-of-mouth: affective pathways through awe and restoration in high-altitude ecocultural tourism using a mixed-methods design

**DOI:** 10.3389/fpsyg.2025.1740267

**Published:** 2026-01-12

**Authors:** Wenguo Liao, Muhammad Shahid Khan, Guangping Liao

**Affiliations:** 1 School of Economics and Management, Aba Teachers College, Wenchuan, China; 2 College of Innovation Management, Suan Sunandha Rajabhat University, Bangkok, Thailand; 3 School of Resources and Environment, Aba Teachers College, Wenchuan, China

**Keywords:** awe, destination appraisal, discrete emotions, ecocultural tourism, high-altitude, restorative experience, S-O-R, word-of-mouth

## Abstract

**Introduction:**

High-altitude ecocultural destinations combine natural grandeur with layered cultural meaning, yet the affective pathways linking tourists’ appraisals to word-of-mouth intention remain underspecified. This study specifies discrete-emotion mechanisms and their ordering within an appraisal–emotion–behavior chain, grounded in the Stimulus–Organism–Response (S–O–R) paradigm.

**Methods:**

A two-phase, text-driven design was implemented. Phase I applied semantic modeling to 23,289 user-generated reviews to derive indicators of three appraisal cues (place identification, perceived cultural heterogeneity, perceived ecological authenticity) as well as awe, restorative experience, and word-of-mouth intention. Phase II tested a theory-driven S–O–R structural equation model on an independent multi-site tourist survey (*n* = 439) from ten destinations on the Northwestern Sichuan Plateau using PLS-SEM with bootstrapped mediation.

**Results:**

The hypothesized dual-pathway architecture was supported. Place identification positively affected awe, whereas perceived cultural heterogeneity and perceived ecological authenticity positively affected restorative experience. Awe preceded and strengthened restoration, and both mediators positively affected word-of-mouth intention. Mediation analyses substantiated appraisal-to-intention transmission via awe for place identification and via restoration for cultural heterogeneity and ecological authenticity, as well as a sequential mediation from awe through restoration to intention.

**Discussion:**

Findings advance appraisal and broaden-and-build accounts by showing that positive affect in nature–culture contexts is not generic but functionally differentiated and ordered. Practically, destination design and interpretation should enhance symbolic elevation to elicit awe and strengthen ecocultural coherence to support restoration—for example through curated viewframes, respectful ritual and vernacular cues, and protected quiet and dark-sky corridors—thereby increasing recommendation intentions while preserving cultural and ecological authenticity.

## 1 Introduction

User-generated content (UGC) on digital platforms shapes how travel narratives circulate by enabling tourists to make sense of their experiences, turn them into narratives, and share them with others, which in turn influences individual meaning-making and collective understandings of destinations (Litvin et al., 2008; Filieri, 2016). In high-altitude ecocultural contexts, remoteness, ecological fragility, and cultural heterogeneity elicit strong emotional responses and symbolic interpretations (Echtner and Ritchie, 1991; Kirillova et al., 2014). When natural grandeur coincides with culturally authentic cues within liminal spatiality, experiential immersion typically increases and self-transcendent emotions, particularly awe, together with restorative psychological states are frequently observed (Kaplan, 1995; Wang, 1999; Keltner and Haidt, 2003; Kirillova et al., 2014). These affective states are proximal antecedents of prosocial behavioral intentions. This includes word-of-mouth (WOM) communication (Hosany et al., 2017; Kim et al., 2022).

Yet as these high-altitude ecocultural destinations expand in popularity and in policy significance, clarifying how visitors’ on-site appraisals channel into discrete emotions and, ultimately, prosocial behaviors has become critical. Worldwide, mountain tourism represented about 9 to 16% of international arrivals in 2019, equivalent to roughly 195 to 375 million visits (FAO and UNWTO, 2023). Within China, northwestern Sichuan’s highland region now functions as a major domestic hub; Ganzi Tibetan Autonomous Prefecture alone registered 41.33 million visits in 2023 (China News Service, 2023). Peak-holiday flows strengthen the trend, with Mount Siguniang receiving 82,946 visitors during May Day 2023, a year-on-year rise exceeding 287% (China Daily, 2023), and inbound arrivals to Jiuzhaigou and Huanglong increasing by 70.6 and 33.6% during May Day 2025 (Sichuan Government, 2025). Current national policies that promote “All for One” tourism and ethnic regional revitalization reinforce this positioning at the nexus of ecological, cultural, and economic objectives (Ministry of Culture and Tourism, PRC, 2022).

Despite rising interest from scholarship and policy, pathways from destination appraisal to restorative experience and prosocial intention remain thinly specified in high-altitude ecocultural contexts (Nanggong and Mohammad, 2020; Qiu et al., 2025). Previous studies have three limitations. One is reliance on prespecified, top-down models that may fail to reflect tourists’ meaning-making (Yoon and Uysal, 2005; Jeongmi and Fesenmaier, 2017). A second limitation is the frequent reduction of emotional responses to valence, a practice that obscures discrete states (e.g., awe and restorative experience) with distinct psychological roles (Hosany et al., 2017; Kim et al., 2022). A third limitation is the lack of integrated, theory-grounded analyses that simultaneously test how place identification, perceived cultural heterogeneity, and ecological authenticity differentially relate to discrete emotions and how those emotions sequentially mediate effects on word-of-mouth intention (Maier et al., 2021; Zhou et al., 2022; Liu et al., 2025).

To address these deficiencies, this study employs a two-phase, bottom-up, text-driven design. In Phase I, semantic topic modeling (Latent Dirichlet Allocation) with community detection is applied to 23,289 user-generated reviews to derive text-based indicators for appraisal-like perceptions, affective states, and word-of-mouth intention. In Phase II, these indicators inform a stimulus–organism–response (S-O-R) structural equation model estimated on an independent survey sample (*n* = 439), grounded in cognitive appraisal theory, attention restoration, and research on self transcendent emotion, to test whether appraisal based perceptions shape interpersonal recommendation intentions through functionally distinct emotional pathways (Lazarus, 1991; Kaplan, 1995; Keltner and Haidt, 2003; Arcangeli et al., 2020).

Accordingly, this study addresses the following research questions:

RQ1. Which appraisal-like perceptual dimensions and affective states emerge from a semantic analysis of tourist narratives in high-altitude ecocultural settings?

RQ2. How do appraisal-like perceptions relate to functionally distinct emotional responses along differentiated pathways?

RQ3. Do the identified emotional responses mediate the association between appraisal-like perceptions and prosocial communication intentions in a staged manner, including a possible sequential relationship among the emotions?

This research offers two theoretical advances in high-altitude ecocultural contexts. First, it supports a dual-pathway, cascading mechanism for word-of-mouth (WOM) intention: place identification operates mainly through awe, whereas cultural–ecological cues operate mainly through restorative experience; awe further strengthens restorative experience, yielding a sequential indirect pathway. Second, it refines the “organism” component of the Stimulus–Organism–Response (S–O–R) model by conceptualizing it as a differentiated and potentially ordered affective process rather than a unitary positive state, thereby clarifying how distinct perceptual cues translate into WOM as a prosocial communication intention via specific emotional routes. The proposed mechanism also has practical relevance for promoting pro-environmental orientations in ecologically sensitive destinations.

## 2 Literature review

### 2.1 Perceptual stimuli and affective dual-pathways: S–O–R perspective

Tourist experiences are shaped by destination appraisals—here understood as place identification, perceived cultural heterogeneity, and perceived ecological authenticity—that supply meaning and relevance to the environment (MacCannell, 1973; Montello, 1993; Hughes, 1995; Gustafson, 2001; Kianicka et al., 2006; Lewicka, 2011b; Li, 2014). Consistent with an appraisal-based view of emotion, these appraisals recruit discrete emotional functions rather than a monolithic affect: awe, a self-transcendent response to perceived vastness and symbolic elevation, and restorative experience, characterized by attentional replenishment and reduced cognitive load in nature (Kaplan, 1995; Keltner and Haidt, 2003).

Framed by the Stimulus–Organism–Response (S–O–R) perspective, destination appraisals (S) elicit awe and restorative experience as organismic states (O), which in turn shape the response (word-of-mouth intention) conceived as a prosocial communication intention to share and recommend one’s experience (Hosany et al., 2017; Wang et al., 2020). The model is anchored in cognitive appraisal theory and attention-restoration accounts, with broaden-and-build providing the motivational bridge from positive emotion to outward social action (Lazarus, 1991; Kaplan, 1995; Fredrickson, 2001).

However, recent research in tourism and hospitality utilizing S–O–R framework has frequently characterized the organismic component through broad affective measures, including general arousal or overall positive affect (Su et al., 2020; Hosany et al., 2021). This approach makes it difficult to distinguish among functionally heterogeneous positive states. The research on awe and restorative experience is substantial, but it has evolved along divergent paths. Studies in nature- and mountain-based tourism define awe as a self-transcendent feeling triggered by perceived immensity and transcendence, while examining its potential behavioral consequences (Keltner and Haidt, 2003; Pearce et al., 2017; Tsaur et al., 2024). The environmental-psychology tradition, conversely, emphasizes restorative experience, highlighting attentional replenishment and psychological recovery in environments marked by coherence and gentle attraction (Kaplan and Kaplan, 1989; Kaplan, 1995). Current tourism research has rarely incorporated these two affective mechanisms into a cohesive theoretical framework and has paid less consideration to their potential temporal sequencing, such as whether awe may act as a precursor to restorative experience. This study introduces a “dual-pathway” cascade model to address the identified limitation: distinct types of perceptual signals are associated with awe and restorative experience, respectively; awe subsequently enhances restorative experience; and collectively, these processes influence tourists’ intention to engage in word-of-mouth communication.

### 2.2 Role of place identification in eliciting awe

Place identification (PI) captures the cognitive recognition of spatial and symbolic markers—iconic scenery, sacred sites, landmarks, and culturally resonant place names—that stabilizes spatial awareness and lends meaning to later emotional responses (Montello, 1993; Gustafson, 2001; Lewicka, 2011b). From an appraisal perspective (Lazarus, 1991), recognising culturally or geographically distinctive features, for example Five-Color Lake and Chonggu Temple, heightens appraisals of extraordinariness, vastness, and symbolic elevation and thereby elicits awe, a self-transcendent emotion aligned with the landscape-sublime tradition (Keltner and Haidt, 2003; Arcangeli et al., 2020; Rivera et al., 2020). High-altitude sacred settings often combine perceived magnitude with moral–spiritual significance, which amplifies the aesthetic and existential tones of awe (Setten and Brown, 2009; Ferriolo, 2019). Accordingly, the following hypothesis is proposed:

*H1:* Place identification has a positive effect on awe.

### 2.3 Perceived cultural heterogeneity as a driver of restorative experience

Perceived cultural heterogeneity (PC) captures visitors’ readings of ethnic plurality, ritual practice, vernacular aesthetics, and customary lifeways at a destination (Sánchez-Rivero and Pulido-Fernández, 2012; Mazanec et al., 2015). In attention–restoration accounts, culturally coherent diversity affords soft fascination: it holds attention with little effort and thickens the meaning of place (Kaplan and Kaplan, 1989; Li, 2014; Mazanec et al., 2015). In high-altitude ecocultural settings, layered narrative cues—prayer rituals, vernacular architecture, seasonal performances—give visitors interpretive handles for engagement and symbolic reappraisal, which supports attentional recovery and affective renewal (Hernández-Mogollón et al., 2018; Yang et al., 2022). Cultural restoration thus emerges from meaning-making and patterned diversity in humanized environments and sits alongside biophysical routes. Accordingly, higher cultural heterogeneity should relate to stronger restorative experience.

*H2:* Perceived cultural heterogeneity positively affects restorative experience.

### 2.4 Perceived ecological authenticity as an affective resource for restorative experience

Perceived ecological authenticity (PE) refers to appraisals of a destination as ecologically intact—pristine, biodiverse, and minimally affected by human intervention—thereby evoking judgments of natural purity and an elemental return to nature (Cohen, 1988; Hughes, 1995; Kianicka et al., 2006). In high-altitude ecocultural contexts, such appraisals signal environmental integrity and enable meaning-laden contact with “unspoiled” landscapes. In line with Attention Restoration Theory, environments with low anthropogenic intrusion and high biophysical coherence and richness are understood to elicit soft fascination, lighten cognitive load, and promote attentional renewal and affective recovery (Kaplan, 1995). In high-altitude ecocultural contexts, conditions of clean air, quiet soundscapes, and wide, undisturbed vistas accord with the mechanism and are associated with greater restorative experience (Zhang et al., 2023; Cheng et al., 2025). On this basis, the following hypothesis is advanced:

*H3:* Perceived ecological authenticity has a positive effect on restorative experience.

### 2.5 Awe as a cognitive–affective bridge to word-of-mouth intention

Awe (AW) characterized as a self-transcendent reaction to perceived vastness and symbolic elevation, serves as the essential link between evaluation and behavior (Keltner and Haidt, 2003). In high-altitude ecocultural environments, the acknowledgment of culturally significant landmarks, sacred geographies, and monumental landscapes enhances perceptions of extraordinariness; consequently, place identification (PI), by emphasizing iconic spatial and symbolic indicators (e.g., Five-Color Lake, Chonggu Temple), intensifies feelings of awe (Setten and Brown, 2009; Ferriolo, 2019).

Beyond its immediate affective impact, awe broadens attention and readiness to “accommodate” experience, laying cognitive–affective groundwork for restorative experience (RE) through soft fascination and reduced cognitive load (Kaplan, 1995; Chirico and Yaden, 2018). Simultaneously, the elevating and memorable nature of awe fosters prosocial communication—a readiness to validate, share, and recommend one’s experience—thereby increasing word-of-mouth intention in both in-person and digital contexts (Van Cappellen and Saroglou, 2012; Xu and Hu, 2024).

Thus, the subsequent hypotheses are posited:

*H4a:* Awe has a positive effect on restorative experience.

*H4b:* Awe has a positive effect on word-of-mouth intention.

*H6a:* Awe positively mediates the effect of place identification on word-of-mouth intention.

### 2.6 Restorative experience as a mediator of cultural and ecological appraisals

Restorative experience (RE) denotes an affective condition of mental recovery, emotional replenishment, and effortless immersion arising in environments that afford soft fascination and psychological detachment (Kaplan, 1995). In line with the broaden-and-build account of positive emotion (Fredrickson, 2001), Restorative experience not only alleviates cognitive fatigue but also promotes prosocial communication—including a readiness to recommend and share experiences as word-of-mouth intention (Wang et al., 2020; Chua et al., 2025).

Two appraisal-based antecedents are anticipated to provoke restorative experience. Perceived cultural heterogeneity—the sensed richness and distinctiveness of ethnic symbols, rituals, vernacular aesthetics, and customary practices—invites meaning-laden engagement and gentle attentional capture, even in unfamiliar settings, thereby supporting attentional replenishment and affective renewal (Hernández-Mogollón et al., 2018; Seyfi et al., 2020). Perceived ecological authenticity—assessments of environmental cleanliness, minimal human interference, and biodiversity—has been associated with psychological restoration; experiences of clean air, tranquil soundscapes, flowing water, and vast alpine vistas diminish cognitive load and promote restorative experience (Tang et al., 2024a; Zhang et al., 2024).

Based on these mechanisms and the study’s sequential perspective, the following hypotheses are proposed:

*H5:* Restorative experience has a positive effect on word-of-mouth intention.

*H6b:* Restorative experience mediates the relationship between perceived cultural heterogeneity and word-of-mouth intention.

*H6c:* Restorative experience mediates the relationship between perceived ecological authenticity and word-of-mouth intention.

*H6d:* Restorative experience mediates the relationship between awe and word-of-mouth intention.

**Figure 1:**
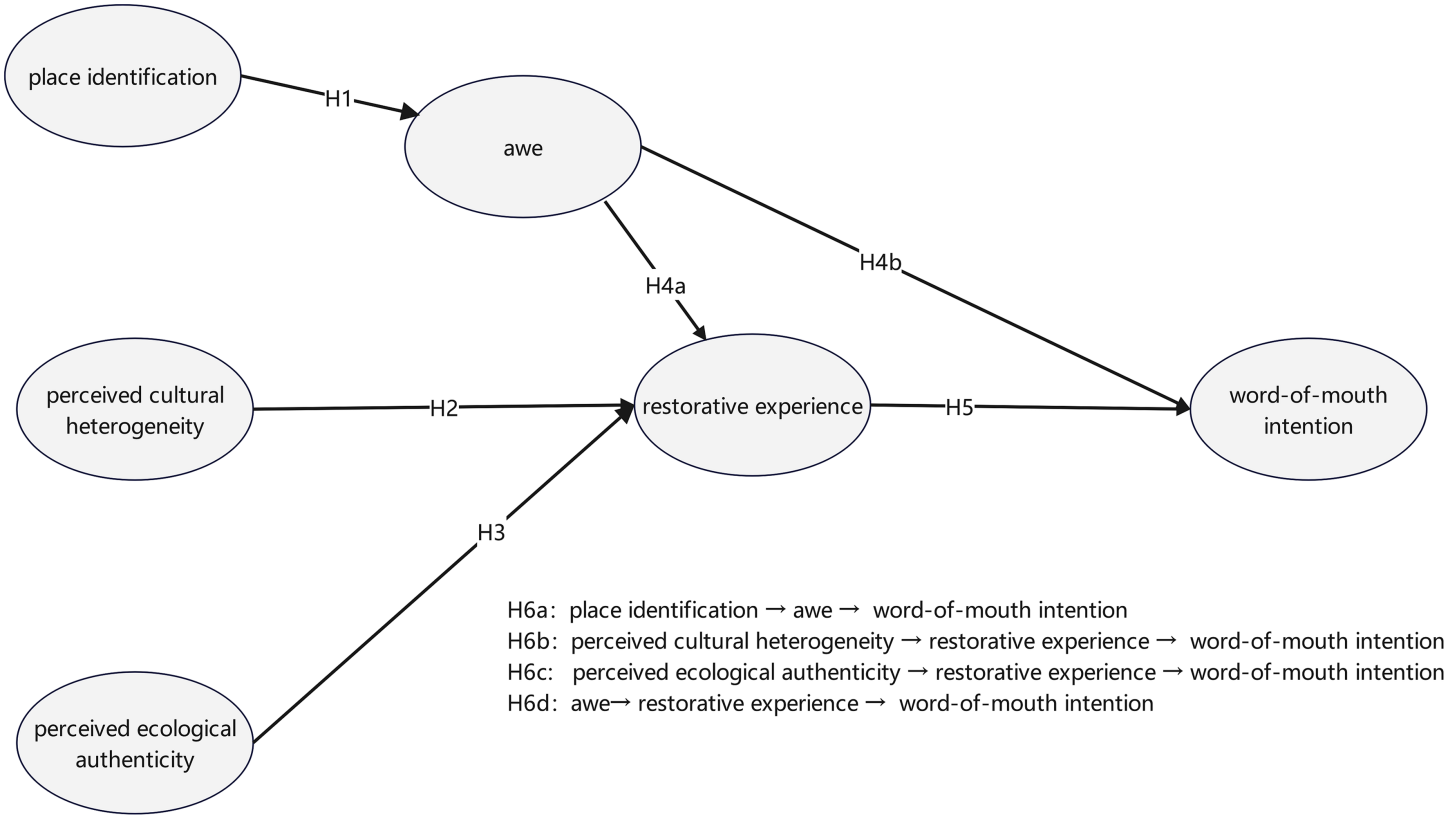
Conceptual model and hypotheses.

In sum, the hypothesized effects compose a sequential appraisal–emotion process culminating in word-of-mouth intention. The resulting conceptual model is shown in Figure 1.

## 3 Materials and methods

An exploratory sequential mixed-methods design was employed. In Phase I, public Ctrip reviews from high-altitude ecocultural destinations were collected and preprocessed; Latent Dirichlet Allocation (LDA) and Louvain community detection were applied to delineate semantic communities aligned with an appraisal–emotion framework (Blei et al., 2001; Blondel et al., 2008; Maier et al., 2021). Credibility is enhanced through a blind human-coding audit and a reflective measurement assessment. In Phase II, an independent-survey operationalization was followed by estimation of a dual-path mediation model via PLS-SEM (SmartPLS 4), with indirect effects tested using BCa bootstrapping (5,000 resamples). PLS-SEM was chosen given reflective specification, complex paths, small-to-medium samples, and non-normal data, consistent with exploratory, prediction-oriented goals (Hair et al., 2022).

### 3.1 Phase I: semantic derivation and validation of text-based measures

#### 3.1.1 Data collection and preprocessing

User-generated textual reviews were sourced from Ctrip, one of China’s leading online travel platforms. By May 2025, 23,289 reviews were collected from ten high-altitude ecocultural destinations on the Northwestern Sichuan Plateau—Siguniang Mountain (5,621), Daocheng Yading (3,000), Jiuzhaigou (3,000), Bipeng Valley (3,000), Gongga Mountain–Hailuogou (3,000), Huanglong Scenic Area (2,999), Hongyuan–Ruoergai Grassland (1,728), Xinduqiao (400), Larung Gar Buddhist Academy (271), and Jiaju Tibetan Village (270). After de-duplication, language/length screening, and text normalization—including regex cleaning, stop-word filtering, synonym normalization, and domain-adapted Jieba tokenization—the analytic corpus comprised 16,420 reviews. All texts were de-identified prior to analysis and processed in accordance with the platform’s terms of use. Under applicable local policy, secondary analysis of public, non-identifiable text does not constitute human-subjects research; no personally identifiable information was collected, stored, or reported.

#### 3.1.2 From semantic clusters to latent constructs

**Figure 2:**
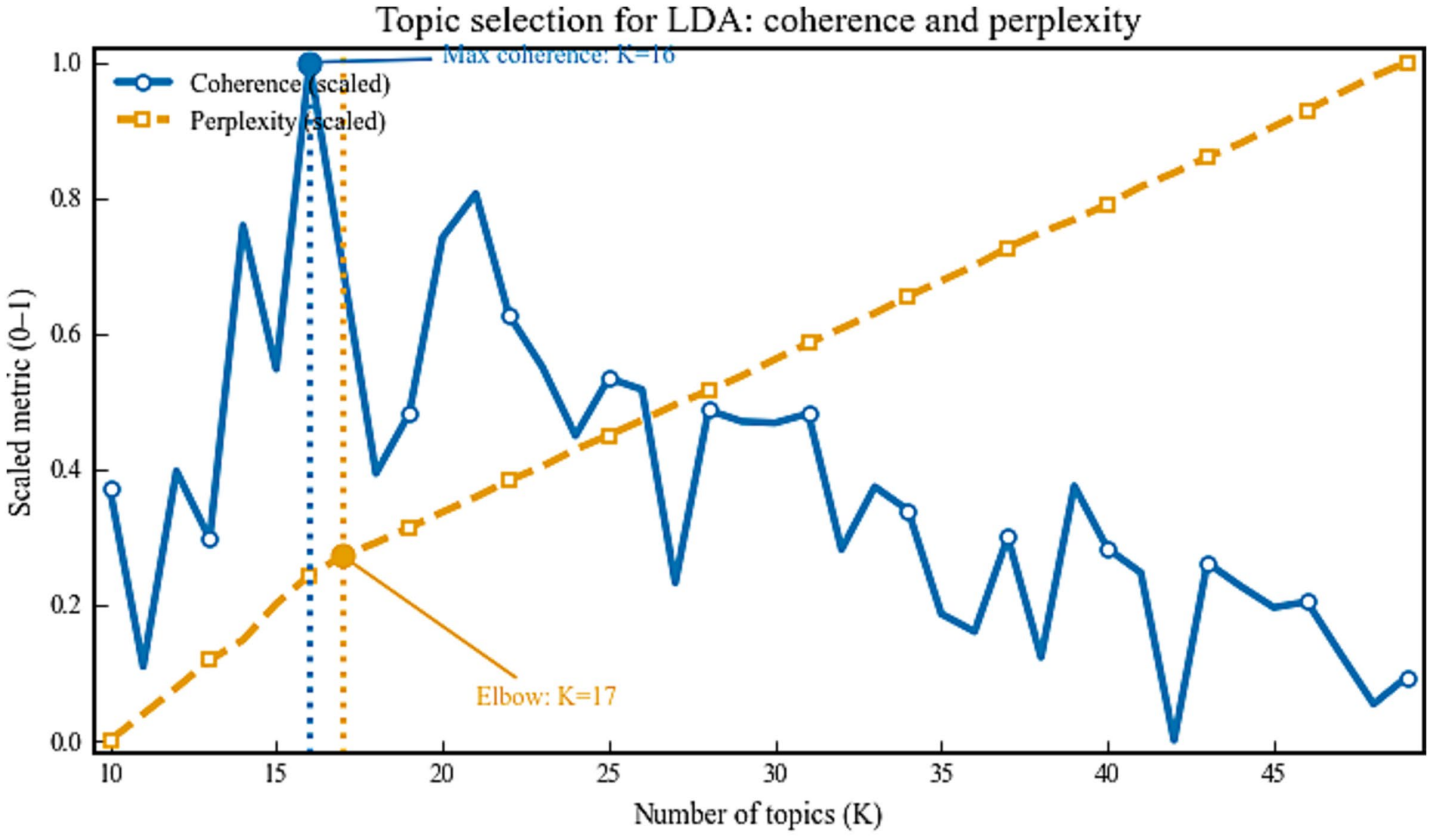
Topic number evaluation via coherence and perplexity.

Latent Dirichlet Allocation (LDA) was used to recover latent thematic structure. The number of topics was selected with a dual criterion that combined the maximum of topic coherence and the elbow of log perplexity, both rescaled to a common axis for comparability (Figure 2). Coherence reached its maximum at K=16 , whereas log perplexity decreased monotonically with K and exhibited an elbow at approximately K=17 . To balance statistical fit with semantic interpretability, K=16 was retained.

To alleviate the granularity and theme duplication inherent in LDA-generated topics, community detection was applied to the topic-keyword network based on term co-occurrence and semantic textual similarity. During this identification phase, semantically related topics are aggregated to higher-level semantic clusters. Aggregated semantic clusters improve understanding of the concepts and help to develop a theory-based construct (Maier et al., 2021). The study applied the Louvain algorithm to the topic keywords’ cosine-similarity (Blondel et al., 2008). Community detection identified seven semantically meaningful communities, which were then inductively interpreted with prominence of the keywords, and linked to latent constructs of tourism and consumer psychological theories (Calheiros et al., 2017; Jacobi et al., 2018; Maier et al., 2021). By integrating computational clustering with expert-guided interpretation, this approach ensures both empirical robustness and theoretical relevance. Seven semantic communities were identified via topic clustering and inductively mapped to theoretical constructs, including perceived ecological authenticity, place identification, perceived cultural heterogeneity, awe, restorative experience, perceived service quality, and word-of-mouth intention (see Table 1 for topic assignments and illustrative keywords).

**Table 1:** Construct development via topic clustering and community detection.

Community	Topic	Construct	Illustrative keywords
Community#1	Topic#8	Perceived ecological authenticity	(1) Snow mountain, glacier, waterfall, forest, virgin forest, lakes, marsh, black forest, lake water, Haizi
			(2) Air, cold air, thin air, fresh, purity, vegetation, ecological environment, ecosystem, natural, nature
			(3) Squirrel, wildlife, green ecology, Glacier rivers, Mountain glacier, Valley glacier
			(4) Ecotourism, primitive society, national forest park, forest park, sea, Caodianzi
Community#2	Topic#6	Place identification	(1) Multicolored pool, Cow Milk Sea, Mirror Lake, Five Flower Sea, Flower Lake, Shuangqiaogou, Dahaizi, Changhai
			(2) Chonggu Temple, Great Sutra Hall, Monk’s quarters, Tang Kexiang, Xiannai Ri
			(3) Tangke, Tangke Town, Zhuoma Beach, Stop at Zhuoma Beach, Shuzhenghai
			(4) Xuebao Ding, Xuebaoding, Xuebao Dinghai, Xuebao Dingmi, Xuebao Ding Xueguang, Youmayon
Community#3	Topic#12	Word-of-mouth intention	(1) Recommend, evaluate, pass from mouth to mouth, Word-of-mouth, praise
			(2) Reputation, great reputation, famous, fame, well-known, popular, ranking
			(3) Favorable comment, the public
Community#4	Topic#5,15	Perceived cultural heterogeneity	(1) The Zang nationality, Tibetans, Qiang ethnic group, Tibetan compatriots, ethnic minority, Chinese nation, nationality
			(2) Temple, lama, lamasery, Prayer flag, religion, religious belief, Religious Policy, Lama Temple
			(3) Folk custom, Folk customs, folk culture, traditional custom, local conditions and customs, National dress, ancient, cultural heritage, cultural background
			(4) Tibetan style, cultural atmosphere, Style Garden, cultural palace, cultural sites, cultural environment, cultural city
			(5) Humanity, cultural exchange, cultural city, Chinese culture, cultural sites
Community#5	Topic#4	Restorative experience	(1) Relax, peaceful, quiet, calm, comfortable, warm, soul, comfortable
			(2) Happy, enjoy, happiness, interesting, leisure, leisurely, fun, play, romantic
			(3) Be reluctant to leave, not letting go, miss, be reluctant to part from, never forget, unforgettable
			(4) Immerse, be immersed in, focus on, to one’s heart’s content, selfless, indulge, crazy, revel in, entertainment
Community#6	Topic#2,9,11, 14	Perceived service quality	Service attitude, quality of service, good service, services and facilities, service level, management level, service center, service station, service area, hotel facilities, environmental hygiene, public health, sanitary facilities, cleanliness, comfort
Community#7	Topic#0,1,3,7,10,13	Awe	(1) Open, vast, broad, wide, spacious, space, living space, a vast territory with a sparse population, mountains and rivers, forest steppe, grassland, green grassland, Hulunbuir Grassland
			(2) Beautiful scenery, beautiful, snow scene, wonderland, graceful, picture scroll, landscape, picturesque landscape, majestic, landscape painting, landscape painting done with splashes of ink, magnificent, blue sky, the blue sky and white clouds
			(3) Shock, heaven, spectacular, amazing, wonder, excited, stimulate, fascination
			(4) Few people, free, freedom, few people in the picture, less people, less car passengers, take advantage of the shortage of people, transparent, free choice

#### 3.1.3 Measurement development and construct operationalization

Candidate indicators (see Table 2) were distilled from tourist narratives via topic modeling and community detection and then aligned with an appraisal–emotion framework to form theory-consistent latent variables (Jacobi et al., 2018; Sanchez-Franco et al., 2019; Ogawa and Saga, 2020; Maier et al., 2021).

**Table 2:** Construct operationalization: subdimensions and measurement items.

Construct	Subdimension label	Measurement item
Perceived ecological authenticity	(1) Primitive natural landscape recognition	PE1: The pristine natural environment, such as snow mountains and forests, gave me a sense of unspoiled nature.
	(2) Sensory perception of natural environment	PE2: I felt the air, climate, and vegetation were pure and ecologically intact.
	(3) Ecological vitality awareness	PE3: The diversity of wildlife here conveyed a strong sense of natural vitality.
	(4) Cultural embedding and primal association	PE4: Being here evoked a sense of connection to primitive or tribal ecological life.
Place identification	(1) Natural landmark recognition	PI1: I can clearly recall the names of unique natural landmarks at this destination, such as Five-Color Lake or Milk Lake.
	(2) Cultural/religious symbol recognition	PI2: The religious or cultural architecture deeply shaped my impression of this place.
	(3) Local toponym cognition	PI3: I can identify and remember distinct local place names, such as Tangke or Zhuoma Beach.
	(4) Sacred highland identity	PI4: Sacred highland mountains like Xuebaoding enhanced my emotional attachment to this destination.
Word-of-mouth intention	(1) Recommendation and sharing intention	WOM1: I am willing to recommend this travel experience to others and share it online.
	(2) Public recognition and social reputation	WOM2: This destination enjoys a strong reputation and social presence among travelers.
	(3) Social influence and peer recognition	WOM3: The public attention toward this place motivates me to talk about it and share my impressions.
Perceived cultural heterogeneity	(1) Ethnic identity difference	PC1: I perceived a strong contrast between the local ethnic identity and my own cultural background.
	(2) Religious cultural contrast	PC2: The religious atmosphere here offered me a culturally distinct experience.
	(3) Traditional lifestyle diversity	PC3: Local customs and daily practices strongly reflected a unique ethnic culture.
	(4) Aesthetic and spatial cultural difference	PC4: The architecture and spatial design demonstrated a rich expression of ethnic aesthetics.
	(5) Historical and identity narrative difference	PC5: I sensed a deep historical narrative and cultural identity embedded in this place.
Restorative experience	(1) Relaxation and peace perception	RE1: I felt completely relaxed and peaceful throughout the trip.
	(2) Positive emotion and well-being	RE2: This trip made me feel joyful and happy.
	(3) Emotional attachment and nostalgia	RE3: I felt deeply attached to the destination and reluctant to leave.
	(4) Immersive and flow experience	RE4: I was fully immersed in the experience, forgetting time and worries.
Awe	(1) Grandiosity and spatial vastness	AW1: The vast natural spaces gave me a strong feeling of grandeur and spatial openness.
	(2) Magnificence and visual aesthetics	AW2: The scenery was spectacular and visually pleasing, like a vivid painting.
	(3) Emotional Awe and reverence	AW3: The grand views inspired awe and emotional elevation within me.
	(4) Tranquility and freedom perception	AW4: The secluded and quiet environment provided me with a deep sense of freedom and peace.

Multidimensional destination appraisals were operationalized as place identification (PI), perceived cultural heterogeneity (PC), and perceived ecological authenticity (PE). Place identification indexes recognition of distinctive natural landmarks, cultural–religious symbols, toponyms, and iconic highland features (Lew and McKercher, 2006; Chen and Rahman, 2018); Perceived cultural heterogeneity denotes sensed contrasts in ethnic identity, religious culture, lifeways, aesthetics, and historical narratives (Sánchez-Rivero and Pulido-Fernández, 2012; Li, 2014; Mazanec et al., 2015); Perceived ecological authenticity reflects appraisals of pristine landscapes, ecological integrity, biodiversity, and primal associations (Belhassen and Caton, 2006; Reisinger and Steiner, 2006; Lau, 2010; Zhang et al., 2018). Emotional states comprised awe—grandiosity, aesthetic elevation, reverence, tranquility, freedom (Lewis, 2003; Benjamin, 2011; Korstanje, 2013; Knudsen et al., 2015),—and Restorative Experience (RE)—relaxation, positive affect, attachment/nostalgia, immersive flow (Korpela et al., 2001; Huang, 2021; Backman et al., 2023; Tang et al., 2024a). The outcome word-of-mouth intention (WOM) captures willingness to recommend, perceived public reputation, and peer-driven recognition (Litvin et al., 2008; Abubakar and Mavondo, 2014; Confente, 2015; Pourfakhimi et al., 2020). Perceived service quality, though present in textual themes, was not retained to maintain a cognitive–emotional appraisal remit.

Content validity was supported through domain-expert confirmation of item–construct alignment; targeted wording refinements were made to improve clarity and eliminate redundancy, producing the final item set for structural analysis. Phase II further evaluated item validity and estimated the structural model in PLS-SEM.

### 3.2 Phase II: measurement validation and hypothesis testing via PLS-SEM

#### 3.2.1 Research instrument

An independent cross-sectional survey operationalized the text-derived constructs from phase I (Table 2) utilizing 7-point Likert scale items.

#### 3.2.2 Sample size

The minimum required sample size (*n* = 385) was calculated using a conservative approach assuming *p* = 0.5, a 95% confidence level, and a ± 5% margin of error, as recommended in previous work (Israel, 1992). This meets the minimum requirements for PLS-SEM, which are based on well-known rules like the “10-times rule” and power-based criteria (Hair et al., 2022).

#### 3.2.3 Data source and sampling

Data were collected via a Wenjuanxing survey fielded 10 May–10 June 2025. Convenience sampling targeted recent tourists to high-altitude destinations (e.g., Jiuzhaigou, Siguniang Mountain, Huanglong). Because affect reports were temporal and tourists were geographically dispersed, the design focused on timeliness and breadth (Etikan et al., 2016; Hair et al., 2022). Participants were provided informed consent, and all procedures were in compliance with the guidelines for the ethical use of information.

## 4 Results

### 4.1 Descriptive statistics

A total of 439 valid responses were analyzed (Table 3). The gender proportion was 53.30% male and 46.70% female. Most of the respondents were younger than 50 years old; the largest age groups were 18–30 years (32.57%) and 31–40 years (30.07%). Most of them were well educated (68.14% undergraduate and postgraduate degree). The monthly income was heterogeneous, including 33.26% for CNY 5,001–8,000 and 5.69% with more than CNY 10, 000.

**Table 3:** Demographic characteristics of respondents (*N* = 439).

Demographic	Level	Frequency	Percentage
Gender	Female	205	46.70%
	Male	234	53.30%
Age	18 ~ 30	143	32.57%
	31 ~ 40	132	30.07%
	41 ~ 50	104	23.69%
	51 ~ 60	29	6.61%
	≥61	31	7.06%
Education	High school graduate or less	36	8.20%
	Associate degree	103	23.46%
	Undergraduate	209	47.61%
	Postgraduate degree	91	20.73%
Monthly income	< CNY 3,000	71	16.17%
	CNY 3,001-5,000	122	27.79%
	CNY 5,001-8,000	146	33.26%
	CNY 8,001-10,000	75	17.09%
	> CNY 10,001	25	5.69%

### 4.2 Common method Bias

To examine the potential impact of common method bias, both variance-based and factor-based diagnostics were utilized. The variance inflation factors (VIFs) of all indicators’ outcomes fell in the range from 1.459 to 2.847, which is within the typical acceptable range of at least 3.3 often recommended for PLS-SEM models (Kock, 2015). In other words, there is no detectable collinearity or method bias. Also, Harman’s single-factor test was performed on the data in SPSS through the unrotated principal component analysis procedure. The first extracted factor accounted for 30.25% of the variance in total, falling short of the established 50% cutoff for factor’s variance (Podsakoff et al., 2003). Overall, this test suggests that common method variance is unlikely to constitute a large threat to validity of the results.

### 4.3 Measurement model assessment

#### 4.3.1 Convergent validity and reliability

Indicator reliability was satisfactory: all standardized loadings met or exceeded 0.70 and were statistically significant (*p* < 0.001) (Hair et al., 2022). Internal consistency was acceptable to strong, with Cronbach’s *α* = 0.777–0.897, and composite reliability (CR) = 0.856–0.924, both above the 0.70 rule-of-thumb (Nunnally and Bernstein, 1994). Convergent validity was evidenced by Average variance extracted (AVE) values between 0.598 and 0.791, each exceeding the 0.50 criterion (Fornell and Larcker, 1981). Taken together, these indices support the adequacy of the measurement model for the structural analyses reported in Table 4.

**Table 4:** Construct reliability and convergent validity.

Construct	Item	Loading	Sig test by bootstrapping	Cronbach’s *α*	CR	AVE
PC	PC1	0.860	***	0.897	0.924	0.708
	PC2	0.875	***			
	PC3	0.815	***			
	PC4	0.855	***			
	PC5	0.800	***			
PI	PI1	0.876	***	0.834	0.889	0.667
	PI2	0.744	***			
	PI3	0.810	***			
	PI4	0.831	***			
PEA	PE1	0.727	***	0.777	0.856	0.598
	PE2	0.778	***			
	PE3	0.823	***			
	PE4	0.763	***			
AW	AW1	0.843	***	0.874	0.913	0.725
	AW2	0.869	***			
	AW3	0.833	***			
	AW4	0.860	***			
RE	RE1	0.798	***	0.870	0.912	0.721
	RE2	0.863	***			
	RE3	0.850	***			
	RE4	0.883	***			
WOM	WOM1	0.884	***	0.868	0.919	0.791
	WOM2	0.892	***			
	WOM3	0.876	***			

*p < 0.050; **p < 0.010; ***p < 0.001.

#### 4.3.2 Discriminant validity

Discriminant validity was assessed with two established diagnostics. As shown in Table 5, for each construct the square root of its average variance extracted (main-diagonal entries) exceeded its correlations with the other constructs, satisfying the Fornell–Larcker criterion (Fornell and Larcker, 1981). In addition, the heterotrait–monotrait ratios reported in Table 6 were all below 0.85 (Henseler et al., 2015), providing further evidence of discriminant validity.

**Table 5:** Discriminant validity (Fornell-Larcker criterion).

Construct	PC	PI	PE	AW	RE	WOM
PC	**0.842**					
PI	0.159	**0.817**				
PE	0.172	0.531	**0.773**			
AW	0.194	0.295	0.284	**0.851**		
RE	0.318	0.357	0.349	0.404	**0.849**	
WOM	0.342	0.269	0.253	0.401	0.458	**0.889**

Main diagonal in bold: squareroot of the AVE significance of correlations.

**Table 6:** Discriminantvalidity (HTMTcriterion).

Construct	PC	PI	PE	AW	RE	WOM
PC						
PI	0.179					
PE	0.206	0.649				
AW	0.215	0.334	0.329			
RE	0.355	0.418	0.419	0.458		
WOM	0.387	0.314	0.304	0.453	0.526	

Thresholds are 0.850 for strict discriminant validity.

### 4.4 Structural model assessment

#### 4.4.1 Direct effects

Global model fit was acceptable: SRMR = 0.063 and NFI = 0.843 satisfied recommended criteria (SRMR < 0.08; NFI > 0.80), indicating adequate overall fit (Henseler et al., 2015).

Within the structural model (see Table 7), place identification (PI) significantly predicted awe (AW; *β* = 0.295, *p* < 0.001), accounting for 8.7% of its variance (R^2^ = 0.087), which constitutes a small but non-trivial effect under Cohen’s benchmarks (R^2^ ≈ 0.02 = small; 0.13 = medium; 0.26 = large) (Cohen, 2013), thereby supporting H1. Perceived cultural heterogeneity (PC; *β* = 0.222, *p* < 0.001) and perceived ecological authenticity (PE; *β* = 0.226, *p* < 0.001) each exerted positive effects on restorative experience (RE), as did awe (AW; *β* = 0.296, *p* < 0.001), jointly explaining 26.9% of the variance in RE (R^2^ = 0.269) and supporting H2, H3, and H4a. Word-of-mouth intention (WOM) was increased by awe (*β* = 0.258, *p* < 0.001) and by restorative experience (*β* = 0.354, *p* < 0.001), yielding R^2^ (WOM) = 0.266; H4b and H5 were accordingly confirmed.

**Table 7:** Direct effects.

Hypothesis	Relationship	Path coefficients (β)	Standard error (SE)	*T* values	*p* values	f^2^	R^2^	Q^2^	Supported
H1	AW ← PI	0.295	0.053	5.255	***	0.095	0.087	0.077	Yes
H2	RE ← PC	0.222	0.042	5.237	***	0.064	0.269	0.196	Yes
H3	RE ← PE	0.226	0.045	4.995	***	0.063			Yes
H4a	RE ← AW	0.296	0.045	6.562	***	0.108			Yes
H4b	WOM ← AW	0.258	0.052	4.988	***	0.076	0.266	0.110	Yes
H5	WOM ← RE	0.354	0.042	8.361	***	0.143			Yes

*p < 0.05; **p < 0.01; ***p < 0.001.

Predictive relevance was evidenced for all endogenous constructs (Q^2^ > 0) (Hair et al., 2022). Effect-size diagnostics further indicated that RE had a medium effect on WOM (f^2^ = 0.143), whereas PI had small-to-moderate effects on RE (f^2^ = 0.108) and on WOM (f^2^ = 0.076). Additional small yet meaningful effects were observed for PC on RE (f^2^ = 0.064), PE on RE (f^2^ = 0.063), and PI on AW (f^2^ = 0.095), consistent with conventional thresholds (0.02 small, 0.15 medium, 0.35 large) (Cohen, 2013).

#### 4.4.2 Mediation roles of awe and restorative experience

Mediation tests were conducted to examine whether destination appraisals shape word-of-mouth intention (WOM) through affective mechanisms. As summarized in Table 8, all prespecified indirect effects reached statistical significance. Place identification exhibited an indirect association with WOM through awe (*β* = 0.076, *p* < 0.001; H6a). Perceived cultural heterogeneity and perceived ecological authenticity were each related to WOM via restorative experience, with indirect effects of *β* = 0.079 (*p* < 0.001) and *β* = 0.080 (*p* < 0.001), respectively (H6b, H6c). A sequential pathway from awe to restorative experience and then to WOM was also significant (*β* = 0.105, *p* < 0.001; H6d). Taken together, the pattern indicates that awe and restoration constitute the principal affective conduits through which environmental appraisals foster advocacy-oriented WOM.

**Table 8:** Indirect effects through mediation paths.

Hypothesis	Mediation path	Indirect effect (*β*)	Standard error (SE)	*T* values	*P* values	Supported
H6a	PI → AW → WOM	0.076	0.022	3.534	***	Yes
H6b	PC → RE → WOM	0.079	0.018	4.307	***	Yes
H6c	PE → RE → WOM	0.080	0.019	4.138	***	Yes
H6d	AW → RE → WOM	0.105	0.018	5.679	***	Yes

*p < 0.05; **p < 0.01; ***p < 0.001.

## 5 Discussion

### 5.1 Main findings

This study integrates large-scale semantic analysis of tourist narratives with text-derived measurement development and theory-guided PLS-SEM to clarify how ecocultural appraisals translate into word-of-mouth intention in high-altitude destinations. Three appraisal-like perceptions—place identification, perceived cultural heterogeneity, and perceived ecological authenticity—were associated with two functionally distinct positive states: awe and restorative experience. The findings support a dual-pathway architecture: place identification primarily elicits awe, whereas cultural heterogeneity and ecological authenticity primarily foster restorative experience. In addition, awe precedes and strengthens restorative experience, producing a sequential affective cascade that further promotes recommendation intentions. Theoretically, these findings enhance S–O–R models by defining the organismic stage as distinct and potentially ordered affective processes instead of a singular, undifferentiated positive affect. Practically, the dual-pathway pattern suggests that sustainable management can be enhanced by experiential designs that integrate symbolic place cues to evoke awe with ecocultural coherence and environmental quality to facilitate restoration, potentially augmenting visitors’ WOM intention.

Semantic analysis identified three perceptual dimensions—place identification, cultural heterogeneity, and ecological authenticity—as well as two functionally distinct affective states, awe and restorative experience, thereby addressing RQ1. These constructs are embedded in a high-altitude ecocultural setting where spiritual geography, ethnocultural diversity, and ecological fragility intersect, and they appear to be expressed and emphasized in ways that differ from more conventional tourism contexts. First, prior work commonly treats place identification as an affective attachment to landscape symbols (Williams and Vaske, 2003; Lewicka, 2011a; Zou et al., 2023). In sacred high-altitude environments, however, place meaning is more strongly reflected in visitors’ engagement with a transcendent symbolic order (e.g., meaning systems carried by sacred mountains), extending symbolic attachment from emotional identification toward an embodied form of spiritual practice. Second, although cultural heterogeneity and ecological authenticity are often discussed separately (Stylidis, 2016; Park et al., 2019), they tend to merge in this context into a perception of “ecocultural integrity,” whereby local rituals, vernacular architecture, and pristine alpine ecosystems are experienced as an inseparable whole. This integrative perception helps explain why the two dimensions jointly relate to restorative experience and also suggests that cultural coherence may function as a restorative resource, complementing attention restoration theory’s emphasis on naturalness (Kaplan, 1995). Finally, emotion research links awe to experiential cues of vastness and sacredness (Keltner and Haidt, 2003; Piff et al., 2015; Zhang and He, 2025), whereas restorative experience is typically associated with tranquil immersion, environmental coherence, and attentional recovery processes (Kaplan, 1995; Jeong, 2024; Tang et al., 2024b). Taken together, the present findings provide a context-sensitive delineation of the semantic profiles of awe and restorative experience in high-altitude ecocultural tourism, strengthening the contextual fit of these emotion constructs.

Distinct perceptual cues corresponded to relatively independent affective routes, thereby addressing RQ2. The results indicate that the association between place identification and awe is more pronounced, whereas cultural heterogeneity and ecological authenticity are more strongly linked to restorative experience, suggesting two separable yet co-occurring emotional pathways. Prior studies note that awe is often connected to experience cues related to sacredness and grandeur (Keltner and Haidt, 2003; Piff et al., 2015), and that immersion in natural environments and perceived environmental coherence can facilitate psychological restoration (Kaplan, 1995; Tang et al., 2024b). Yet in tourism research these responses have frequently been examined in isolation or implicitly folded into a single positive-affect dimension in empirical models. In contrast, the present study suggests that a single high-altitude destination encounter may involve two functionally different positive states: awe tends to align with symbolic sublimity and sacred cues, while restorative experience aligns more closely with tranquil immersion and culturally–ecologically coherent settings. This pattern is consistent with the view that emotions can be differentiated by function rather than valence (Chirico and Yaden, 2018; Lucht and Van Schie, 2024). The co-occurrence of these pathways further implies that high-altitude destinations may simultaneously elicit complementary positive emotions, offering a more fine-grained lens for understanding the multiplicity of affective experience in tourism contexts.

RQ3 can be clarified through the mediational patterns. The results show that the effect of place identification on word-of-mouth intention is primarily transmitted through awe, whereas the effects of cultural heterogeneity and ecological authenticity operate mainly through restorative experience (Kaplan and Kaplan, 1989; Hosany and Gilbert, 2010; Prayag et al., 2017). In addition, the model reveals a directional path from awe to restorative experience, such that awe not only carries its own indirect effect but may also contribute to a cascaded mediation by strengthening restoration, thereby increasing the overall mediated influence. This pattern accords with broaden-and-build reasoning, whereby self-transcendent emotions may broaden cognitive–affective resources and create more favorable conditions for subsequent recovery (Fredrickson, 2001; Shiota et al., 2007). Compared with approaches that treat positive affect as a unitary mediator, the present results more clearly specify how different perceptual cues are channeled through distinct emotional functions to shape word-of-mouth intention in high-altitude tourism. They also suggest that awe may serve as a precursor state that participates in the formation of advocacy-oriented responses via restorative experience. In this sense, an “awe–restoration” cascade may provide a supplementary interpretive framework for understanding the dynamic affective pathways of complex tourism experiences.

Previous applications of the stimulus–organism–response (S–O–R) framework in tourism and hospitality have frequently operationalized the organismic component via broad affective indices such as general arousal or PAD-based dimensions, which risk conflating functionally distinct positive emotions (Su et al., 2020; Hosany et al., 2021). Conversely, recent studies in nature-based and mountain tourism are starting to regard awe as a distinct self-transcendent emotion with particular antecedents and behavioral outcomes (Pearce et al., 2017; Tsaur et al., 2024). This study enhances the S–O–R model by illustrating that the organismic phase in ecocultural destinations is not a singular affective state but a structured system of dual pathways: place identification predominantly elicits awe, whereas perceived cultural heterogeneity and ecological authenticity collaboratively promote restorative experience. These pathways are not autonomous; awe precedes and enhances restoration, demonstrating a sequential affective cascade that surpasses the notion of uniform positive arousal. This dynamic architecture provides a more detailed and theoretically sound description of the S–O–R mechanism in high-altitude settings where symbolic sublimity and ecocultural coherence together influence advocacy intentions.

### 5.2 Theoretical implications

The research enhances the S–O–R model of tourism behavior through its analysis of how tourists convert their ecocultural appraisals into tourists’ word-of-mouth intentions. First, whereas prior research often operationalizes the organism component as a general positive affective state (Mehrabian and Russell, 1974; Su et al., 2020; Hosany et al., 2021), this finding indicate that organismic responses can be differentiated into two functionally distinct states, forming a dual-pathway structure. Place identification is more closely associated with awe, whereas perceived cultural heterogeneity and perceived ecological authenticity are more closely associated with restorative experience (Kaplan, 1995; Keltner and Haidt, 2003). Second, the results further suggest that awe tends to precede restorative experience. This implies that within a single tourism encounter, appraisal-evoked self-transcendent emotion may occur earlier and connect to subsequent attention-recovery processes (Kaplan, 1995; Fredrickson, 2001; Shiota et al., 2007). Overall, these patterns indicate that different appraisal dimensions may influence word-of-mouth intention through differentiated—and potentially temporally ordered—affective processes, offering a more fine-grained characterization of the organismic stage in S–O–R models.

### 5.3 Practical implications

Drawing on evidence from ten high-altitude ecocultural destinations on the Northwestern Sichuan Plateau, three management priorities emerge within a dual-emotion S–O–R framework. First, branding and on-site interpretation should consistently cue the three perceptual dimensions—place identification, cultural heterogeneity, and ecological authenticity. At Jiuzhaigou and Daocheng, for example, symbolic framing strengthens identity and recognition, while Tibetan vernacular elements and alpine purity signal heterogeneity and authenticity. Second, experience design should deliberately elicit awe and restoration through elevated viewpoints, ritual/communal spaces, and quiet sanctuary zones that broaden attention and support affective recovery. Third, restorative settings should ensure spatial coherence, credible authenticity cues, and multisensory consonance, given the observed association between restorative experience and word-of-mouth intention. Although derived from the plateau context, these principles are transferable to other high-mountain ecocultural regions (e.g., the Himalayas, Andes, Carpathians).

### 5.4 Limitations and future research directions

This study has several limitations. First, because the data are cross-sectional, temporal ordering and causal effects among appraisals, emotions, and behavioral intentions cannot be established. Therefore, the proposed sequence in which awe precedes restoration should be interpreted cautiously and verified in future research using longitudinal designs (e.g., experience sampling) or experiments (e.g., priming cultural symbols and ecological cues via controlled imagery or VR). Second, the current S–O–R specification is largely unidirectional and does not test possible feedback processes (e.g., whether restorative experience strengthens place identification). It also foregrounds positive affect while leaving negative or mixed emotions that may arise in ecologically fragile or culturally sensitive settings (e.g., eco-anxiety or cultural alienation) outside the model; cross-lagged and mixed-emotion perspectives may help capture these dynamics. Third, the perceptual dimensions were derived from a high-altitude ethnocultural context in which natural scenery, cultural practices, and spiritual symbolism are often co-articulated, which may limit transferability to lowland, urban, or less symbolically salient destinations; cross-context replications are needed to assess external validity. Finally, the study did not examine cultural and individual boundary conditions. Interpretations of “ecological authenticity” and “awe” may vary across cultural meaning systems (e.g., East Asian versus Western emphases), and individual traits such as ecological values, prior high-altitude travel experience, and spirituality may condition appraisal–emotion links. Future studies could test these moderators using multi-cultural comparisons, multi-group analyses, or moderation (including moderated-mediation) models.

## Data Availability

The datasets presented in this study can be found in online repositories. The names of the repository/repositories and accession number(s) can be found at: https://osf.io/ub6mv/files.
